# Peptidylarginine Deiminase Inhibitors Reduce Bacterial Membrane Vesicle Release and Sensitize Bacteria to Antibiotic Treatment

**DOI:** 10.3389/fcimb.2019.00227

**Published:** 2019-06-27

**Authors:** Uchini S. Kosgodage, Paul Matewele, Giulia Mastroianni, Igor Kraev, Dominik Brotherton, Brigitte Awamaria, Anthony P. Nicholas, Sigrun Lange, Jameel M. Inal

**Affiliations:** ^1^Cellular and Molecular Immunology Research Centre, School of Human Sciences, London Metropolitan University, London, United Kingdom; ^2^School of Biological and Chemical Sciences, Queen Mary University of London, London, United Kingdom; ^3^School of Life, Health and Chemical Sciences, The Open University, London, United Kingdom; ^4^Bioscience Research Group, Extracellular Vesicle Research Unit, School of Life and Medical Sciences, University of Hertfordshire, Hatfield, United Kingdom; ^5^Department of Neurology, University of Alabama at Birmingham, Birmingham, AL, United States; ^6^Tissue Architecture and Regeneration Research Group, School of Life Sciences, University of Westminster, London, United Kingdom

**Keywords:** outer-membrane vesicles (OMVs), peptidylarginine deiminase (PAD), deimination/citrullination, antibiotic sensitivity, *E. coli* VCS257, *S. aureus* subsp. *aureus* Rosenbach

## Abstract

Outer membrane and membrane vesicles (OMV/MV) are released from bacteria and participate in cell communication, biofilm formation and host-pathogen interactions. Peptidylarginine deiminases (PADs) are phylogenetically conserved enzymes that catalyze post-translational deimination/citrullination of proteins, causing structural and functional changes in target proteins. PADs also play major roles in the regulation of eukaryotic extracellular vesicle release. Here we show phylogenetically conserved pathways of PAD-mediated OMV/MV release in bacteria and describe deiminated/citrullinated proteins in *E. coli* and their derived OMV/MVs. Furthermore, we show that PAD inhibitors can be used to effectively reduce OMV/MV release, both in Gram-negative and Gram-positive bacteria. Importantly, this resulted in enhanced antibiotic sensitivity of both *E. coli* and *S. aureus* to a range of antibiotics tested. Our findings reveal novel strategies for applying pharmacological OMV/MV-inhibition to reduce antibiotic resistance.

## Introduction

Outer membrane vesicles (OMVs), and membrane vesicles (MVs), are released from Gram-negative and Gram-positive bacteria and participate in bacterial communication, facilitating the transfer of cargo molecules (Dorward and Garon, 1990; Li et al., 1998; Fulsundar et al., 2014; Jan, 2017; Toyofuku et al., 2019). OMVs are released in greater abundance from Gram-negative than Gram-positive bacteria, are crucial for bacterial survival and form part of the stress response (McBroom and Kuehn, 2007; Macdonald and Kuehn, 2013; Jan, 2017). Research on bacterial OMVs has grown rapidly in recent years, including their use as bioengineered drug delivery vehicles (Gujrati et al., 2014; Bitto and Kaparakis-Liaskos, 2017) and in vaccine development (Gaillard et al., 2014; Choi et al., 2015; Alves et al., 2016; Raeven et al., 2016; Wang et al., 2017).

Peptidylarginine deiminases (PADs) are a group of calcium-activated enzymes that are preserved throughout phylogeny from bacteria to mammals and catalyze the post-translational deimination/citrullination of arginine residues to citrulline, causing structural, and functional changes in target proteins (Vossenaar et al., 2003; Wang and Wang, 2013; Witalison et al., 2015; Magnadóttir et al., 2018). Five mammalian PAD isozymes have been identified which participate in physiological and pathophysiological processes, including autoimmune and neurodegenerative diseases, cancer and sepsis (Wang and Wang, 2013; Witalison et al., 2015; Kosgodage et al., 2017, 2018; Lange et al., 2017; Biron et al., 2018; Costa et al., 2018). Recent studies have highlighted novel PAD-mediated mechanisms of extracellular vesicle (EV) release in eukaryotic cells (Kholia et al., 2015; Kosgodage et al., 2017, 2018; Gavinho et al., 2019) but a link to conserved mechanisms in bacterial OMV/MV release has hitherto not been made. While several PAD isozymes, with different preferences for target proteins, are present higher in phylogeny, in bacteria only one PAD form has been described. For example in *Porphyromonas gingivalis*, a Gram-negative bacterium, the association of PAD and its citrullinome has been linked to neo-epitope generation in oral cavity disease and rheumatoid arthritis (Maresz et al., 2013; Gully et al., 2014; Stobernack et al., 2016; Bereta et al., 2019). However, although an arginine deiminase (AD) has been identified in the *Eschericia coli* genome (GenBank: EDV68547.1), also a Gram-negative bacterium, no significant data is available to confirm the presence of an associated citrullinome. Furthermore, an AD has also been identified in *Staphylococcus aureus* (GenBank: BBA25170.1), a Gram-positive bacterium.

Our previous studies established that EV release from cancer cells is largely PAD-driven, can be effectively inhibited using pharmacological PAD inhibitors and that such inhibition sensitizes cancer cells to chemotherapy (Kholia et al., 2015; Kosgodage et al., 2017, 2018). Therefore, we set out to investigate if this could be a phylogenetically conserved mechanism and in the same vein, be exploited to sensitize bacteria to antibiotics.

The role of OMVs in biofilm formation and in protecting biofilms via adsorption of antimicrobial agents has indeed been previously recognized (Schooling and Beveridge, 2006; Manning and Kuehn, 2011; Toyofuku et al., 2019). Thus, application of OMV inhibition could potentially lower resistance to antibiotics and be useful in minimizing multi-drug resistance associated with antibiotic treatment.

Using a range of antibiotics, we determined the effect of several PAD-specific inhibitors on changes in OMV/MV release and on antibiotic sensitivity of Gram-negative (*E. coli* VCS257) and Gram-positive bacteria (*S. aureus* subsp. *aureus* Rosenbach).

The PAD inhibitors tested were first generation pan-PAD inhibitor Cl-amidine (Luo et al., 2006), second generation pan-PAD inhibitor BB-Cl-amidine (Knight et al., 2014), PAD2 inhibitor AMF30a (Muth et al., 2017), and PAD4 inhibitor GSK199 (Lewis et al., 2015). The following range of antibiotics was tested in combination with the OMV/MV inhibitors: **(i)** Colistin (Polymyxin E), which acts on the lipoglycans and endotoxins of the Gram-negative bacterial cell membrane (Falagas et al., 2005; Livermore et al., 2011; Yahav et al., 2012;Yu et al., 2019); **(ii)** Vancomycin, which alters the permeability of the cell membrane and selectively inhibits ribonucleic acid synthesis (Watanakunakorn, 1984). It is effective against Gram-positive bacteria, including *Staphylococcus, Streptococcus, and Listeria* and prescribed for serious skin, blood-borne and joint infections as well as meningitis caused by methicillin-resistant *Staphylococcus aureus* (MRSA) (Ng et al., 2014); **(iii)** Rifampicin, which inhibits DNA-dependent RNA polymerase activity, suppressing the initiation of RNA synthesis (Campbell et al., 2001). It is effective against a broad spectrum of bacteria, mainly Gram-positive cocci (van Ingen et al., 2011); **(iv)** Kanamycin, which binds to the bacterial 30S ribosomal subunit, causing misreading of t-RNA and inhibition of bacterial protein synthesis (Hoerr et al., 2016). It is active against most Gram-negative bacteria and some Gram-positive bacteria (Salian et al., 2012); **(v)** Erythromycin, which inhibits bacterial protein synthesis by binding to bacterial 50S ribosomal subunits (Ianaro et al., 2000). It is effective against Gram-positive bacteria including *Staphylococci, Streptococci* and *Pneumococci* and Gram-negative sporing and non-sporing gut anaerobes, such as *E. coli* (Jelić and Antolović, 2016).

Here we show that OMV/MV release can be regulated via PAD-mediated pathways both in *E. coli* VCS257 and *S. aureus* subsp. *aureus* Rosenbach and that this can be exploited to enhance antibiotic effectivity of selected antibiotics in both Gram-negative and Gram-positive bacteria. Furthermore, in *E. coli* we identified deiminated/citrullinated proteins both in the bacterial cells and in derived OMVs, indicative of bacterial communication via lateral transfer of deiminated proteins.

## Materials and Methods

### Preparation of Outer Membrane Vesicles (OMVs) and Membrane Vesicles (MVs)

*E. coli* (VCS257, Agilent, La Jolla, CA) and *S. aureus* subsp. *aureus* Rosenbach (ATCC 29247; CDC73-57501) cultures were grown for 24 h at 37°C (static culture). The growth phase before vesicle isolation was exponential, as assessed by optical density (OD600) before overnight incubation and for 4 h the following day, to ensure that bacteria were in log phase; the volume of the cultures was 20 ml. For OMV/MV-associated experiments, the bacterial growth medium (Luria-Bertani (LB) broth) and Dulbecco's phosphate buffered saline (DPBS)S were pre-treated before use by ultracentrifugation at 100,000 g (SW60Ti rotor, Beckmann L60 ultracentrifuge) for 24 h, to ensure that the medium used was minimally contaminated with extracellular vesicles (EVs).

The OMV/MVs were isolated from the supernatant of the bacterial culture medium as follows: The supernatant was initially centrifuged once at 400 *g* (F-34-6-38 rotor, Eppendorf 5804, U.S.A.) for 10 min to remove the cells. Thereafter, the supernatant was centrifuged at 4,000 *g* (F-34-6-38 rotor, Eppendorf 5804) for 1 h at 4°C to remove cell debris. The resultant supernatant was then centrifuged at 100,000 *g* (SW60Ti rotor, Beckmann L60 ultracentrifuge, Beckman Coulter, U.S.A.) for 1 h at 4°C for isolation of OMVs. The isolated OMV/MV pellet was then resuspended in Dulbecco's phosphate-buffered saline (DPBS; ultracentrifuged and sterile filtered using a 0.22 μm filter) and filtered through an 0.45 μm filter before the second ultracentrifigation step at 100,000 *g* for 1 h at 4°C. The resulting OMV/MV pellet was thereafter resuspended in 100 μl sterile filtered (0.22 μm) DPBS and the isolated OMV/MV pellets were either used immediately, or stored at −80°C for further experiments.

### Nanoparticle Tracking Analysis (NTA) of OMV/MV

For NTA analysis, isolated OMV and MV pellets, respectively, prepared as described above, were resupended in 100 μl sterile filtered DPBS and then diluted 1/200 before quantification to assess vesicle size, based on Brownian motion, using the Nanosight LM10, with a 405 nm diode laser (Malvern, U.K.). Numbers of particles per frame were kept at approximately 30, and five individual 60 s videos were recorded using a sCMOS camera for each sample to create the respective size distribution histograms. OMVs/MVs were further characterized by transmission electron microscopy (TEM) and Western blotting as described below.

### Transmission Electron Microscopy Imaging

A suspension of isolated OMVs/MVs (1.4 × 10^8^ vesicles/ml) was used for TEM imaging. Mesh copper grids were prepared with glow discharged carbon support films and 10 μl of OMV/MV samples applied to the grid and incubated for 2 min. The grids were then washed five times with 50 μl of 1% aqueous uranyl acetate. The last drop was left to incubate on the grid for 1.5 min before being wicked off by torn filter paper. Grids were left to dry for 5 min before being viewed. Micrographs were taken with a JEOL JEM 1230 transmission electron microscope (JEOL, Japan) operated at 80 kV at a range of magnification mainly around a magnification of 80,000–100,000. Digital images were recorded on a Morada CCD camera (EMSIS, Germany) and processed via iTEM (EMSIS, Germany).

### Western Blotting for Membrane Vesicle Characterization

Protein was isolated from the OMV/MV pellets using Bacterial Protein Extraction Reagent (B-PER, ThermoFisher Scientific, U.K.), pipetting gently and shaking the pellets on ice for 2 h, where after samples were centrifuged at 16,000 *g* at 4°C for 20 min and the resulting supernatant collected for protein analysis. Samples were electrophoresed under reducing conditions by SDS-PAGE on 4–20% TGX gels (BioRad, U.K.), followed by semi-dry Western blotting. Membranes were blocked in 5% BSA in TBS-T at RT for 1 h. The membranes were incubated overnight at 4°C with the anti-OmpC (Outer-membrane protein C antibody; orb6940, Biorbyt, U.K.; diluted 1/1,000 in TBS-T). The membranes were washed 3 × 10 min in TBS-T, incubated for 1 h in anti-rabbit-HRP conjugated secondary antibody (BioRad, U.K.) at RT, followed by visualization using ECL (Amersham, U.K.) and the UVP BioDoc-ITTM System (U.K.).

### Immunoprecipitation (IP) and LC-MS/MS Analysis of Deiminated and PAD4 Bound Proteins From *E. coli* VCS257 Cells and Derived OMVs

To determine the presence of deiminated/citrullinated proteins and PAD4 bound proteins from *E. coli* VCS257 and derived OMVs, protein extracts were prepared using Bacterial Protein Extraction Reagent (B-PER, ThermoFisher Scientific, U.K.), according to the manufacturer's instructions. In brief, bacterial cells and OMVs were centrifuged at 5,000 g for 10 min to obtain a pellet. Two microliter of lysozyme and 2 μL of DNase I per 1 mL of B-PER Reagent was added along with EDTA-free protease inhibitors (ThermoFisher Scientific). Four mL of B-PER Reagent per gram of cell pellet was added. The suspension was pipetted up and down until it was homogeneous. The suspension was incubated for 10–15 min at room temperature. The lysate was centrifuged at 16,000 *g* for 5 min to separate soluble proteins from the insoluble proteins. Proteins were thereafter immunoprecipitated, using the Catch and Release® v2.0 Reversible Immunoprecipitation System (Merck, U.K.), according to the manufacturer's instructions, in conjunction with the pan-deimination F95 (MABN328, Merck) antibody (both for *E. coli* and derived OMVs) or the PAD4 (ab96758, Abcam) antibody (for *E. coli* cells only). The F95 pan-deimination specific antibody has been developed against a deca-citrullinated peptide and specifically detects proteins modified by citrullination (Nicholas and Whitaker, 2002). F95 or PAD4 bound proteins, respectively, were eluted from the columns according to the manufacturer's instructions (MERCK), using the supplied elution buffer (non-reducing or a reducing elution buffer, which was supplemented with 5% beta-mercapthoethanol) and thereafter further analyzed by Western blotting, under reducing conditions, and by liquid chromatography-mass spectrometry (LC-MS/MS).

### Western Blotting Analysis of Citrullinated/Deiminated Proteins From *E. coli* VCS257 Cells and Derived OMVs

In order to compare protein profiles of deiminated proteins between *E. coli* and *E. coli* OMVs, total protein lysates and IP protein eluates were subjected to Western blot analysis. Briefly, samples were boiled for 5 min at 100°C in 2x Laemmli sample buffer (BioRad, U.K.). Protein (20 μg per sample) was separated by SDS-PAGE using 4–20% Mini-Protean TGX protein gels (BioRad, U.K) and transferred to nitrocellulose membranes. Even loading was assessed using Ponceau S staining (Sigma, U.K.) and membranes were then blocked in 5% bovine serum albumin (BSA) in Tris buffered saline with 0.1% Tween20 (TBS-T) for 1 h, followed by incubation at 4 °C overnight with the primary antibodies: pan-deimination antibody F95 (1:2,000; MABN328), PAD2 (1:1,000; ab50257), PAD3 (1:1,000; ab169479) and PAD4 (1:1,000; ab96758), respectively. Membranes were washed three times in TBS-T, incubated at room temperature for 1 h with HRP-conjugated secondary antibodies: anti-mouse IgM and anti-rabbit IgG, respectively (both 1:4,000; BioRad, U.K.), followed by six 10 min washes in TBS-T, before visualization using ECL (Amersham, U.K.) and the UVP BioDoc-IT™ System (U.K.).

### Liquid Chromatography-Mass Spectrometry (LC-MS/MS) Analysis

For identification of deiminated/citrullinated proteins and PAD4 bound proteins, respectively, in *E. coli* cell lysate and OMV lysates, the F95 and PAD4 bound eluates were subjected to LC-MS/MS analysis, which was carried out at the Proteomics Service at the Barts Cancer Institute (Queen Mary University, U.K.). For identification of protein hits the peak list files were submitted to in-house Mascot (Matrix Science).

### Effects of PAD Inhibitors on Bacterial OMV/MV Release and Cell Viability

*E. coli* and *S. aureus* were cultivated using EV-free Müeller-Hinton broth for 24 h. Culture condition were as follows: An inoculate of 0.1 ml of bacteria were grown at exponential phase overnight, as assessed by OD600; the volume of the cultures was 20 ml. The cells were washed using DPBS at 4,000 g for 10 min and seeded in triplicate in micro centrifuge tubes. The PAD inhibitors were added in triplicates and incubated for 1 h at 37°C as follows: PAD2 inhibitor AMF30a (5 μM), PAD4 inhibitor, GSK199 (10 μM), pan-PAD inhibitor Cl-amidine (50 μM), and BB-Cl-amidine (5 μM). Cell viability of bacteria in the presence of the different PAD inhibitors was assessed by counting the number (no) of surviving bacterial colonies on the plates. The no. of colonies (viable cells), inoculating volume and dilution factor of the bacterial culture used was used to calculate the viable cells in colony forming unit (cfu/ml) (cfu/ml = (no. of colonies x dilution factor)/volume of culture plate). OMV/MV isolation following treatment was carried out as described above and changes in OMV/MV release were assessed by quantifying numbers of OMVs/MVs by NTA analysis using the Nanosight LM10 as described above. The experiment was repeated three times and replicate histograms were averaged.

### Disc Diffusion Test

*E. coli* VCS257 and *S. aureus* subsp. *aureus* Rosenbach nutrient agar plates were prepared and sterile paper disks were soaked in the PAD inhibitors at the same concentrations as before. Culture medium was 10 ml, log growth phase of bacteria was assessed by OD600, and the inoculum concentration was 0.1 ml. Discs were impregnated with the antibiotics (all from Sigma-Aldrich) at the following concentrations: colistin (10 μg/ml), rifampicin (15 μg/ml), erythromycin (15 μg/ml), kanamycin (1,000 μg/ml), and vancomycin (5 μg/ml). The inhibitor discs were placed in the middle of the agar plates, while the antibiotic discs were placed equi-distant to the respective inhibitor discs to be tested. The Kirby-Bauer test was used to assess the zone of inhibition after 24 h. Supplementary Figure 4 shows the agar plates containing the disks, following completion of the test.

### MIC Measurement of Colistin and Vancomycin in *E. coli* VCS257 and *S. aureus* subsp. *aureus* Rosenbach

MIC values for colistin and vancomycin were tested for *E. coli* and *S. aureus*, respectively. *E. coli* and *S. aureus* suspensions were prepared in Müller Hinton Broth according to Iqbal et al. (2013). For *E. coli*, the concentration of colistin was based on previously published MIC values for colistin, ranging between 2 and 16 μg/ml (Moskowitz et al., 2010; Rojas et al., 2017), and here this range was further expanded between 0.015 μg/ml to 64 μg/ml. MIC values for vancomycin have previously been recorded in the range of 1.5–2 μg/ml (Maclayton et al., 2006; Kshetry et al., 2016; Goldstein et al., 2018) and in some studies a MIC value of 8 μg/ml and higher has been reported (Alam et al., 2014; Lepe et al., 2014). Therefore, for *S. aureus* a MIC confirmation test starting from a concentration of 8 μg/ml was carried out here and thereafter the MIC was considered accordingly. The doubling dilution method was used to obtain triplicates of concentrations used for the antibiotics and a triplicate of control omitting antibiotic was also prepared. The plates were incubated overnight at 37°C and the MIC values of antibiotic treated control wells were compared to wells where PAD inhibitors were applied in combination with the antibiotics.

### Statistical Analysis

The histograms and graphs were prepared and statistical analysis was performed using GraphPad Prism version 8 (GraphPad Software, San Diego, U.S.A.). One-way ANOVA and *t*-test analysis were performed, followed by Tukey's *post-hoc* analysis. Significant differences were considered as *p* ≤ 0.05 and histograms represent mean of data, with error bars representing standard error of mean (SEM).

## Results

### Characterization of Bacterial OMVs/MVs

**Figure 1:**
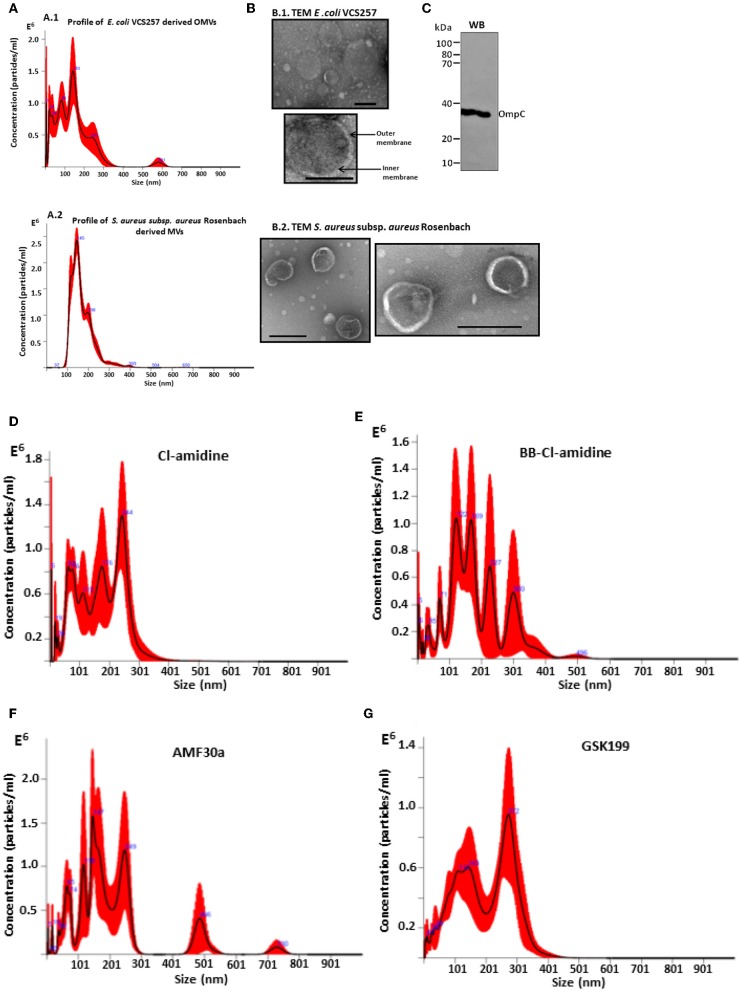
Characterization of *E. coli* VCS257 OMVs and *S. aureus* subsp. *aureus* Rosenbach MVs using NTA, TEM and Western blotting analysis**. (A)** NTA curves, obtained by Nanosight analysis, showing OMVs released from *E. coli* **(A1)** and *S. aureus* **(A2)** under standard conditions; **(B)** Negative stain TEM micrographs of *E. coli* OMVs **(B1)** show the presence of a poly-dispersed sample ranging in size from mainly 20 nm to 320 nm (scale bars represent 200 nm), including vesicles showing inner and outer membranes for *E.coli* VCS257 OMVs **(B1)** and MVs from *S. aureus* subsp*. aureus* Rosenbach **(B2)**. **(C)** OMV-specific marker OmpC verified by Western blotting. **(D–G)** NTA profiles of OMVs released from *E. coli* VCS257 following PAD inhibitor treatment. OMV release profile from *E. coli* treated for 1 h with PAD inhibitors as follows: **(D)** Cl-amidine (first generation pan-PAD inhibitor; 50 μM); **(E)** BB-Cl-amidine (second generation pan-PAD inhibitor; 5 μM); **(F)** AMF30a (PAD2 inhibitor; 5 μM); **(G)** GSK199 (PAD4 inhibitor; 10 μM).

A poly-dispersed population of OMVs was verified by nanoparticle tracking (NTA) analysis, with the majority of vesicles released in the 20–300 nm range for *E. coli* VCS257, and similarly in the 37–300 nm range for *S. aureus* subsp. *aureus* Rosenbach (Figures 1A1, 2). This was further confirmed by transmission electron microscopy, assessing overall morphology and providing average size estimations (Figures 1B1, 2). Morphological analysis by TEM revealed OMVs, including with inner and outer membranes visible, for *E. coli* (Figure 1B1) and typical MVs for *S. aureus* (Figure 1B2). Western blotting verified the presence of the OMV-specific marker OmpC (Figure 1C). Changes in OMV size distribution after PAD-inhibitor treatment in *E. coli* was reflected in a change in NTA profile as shown in Figures 1D–G and further discussed below.

**Figure 2:**
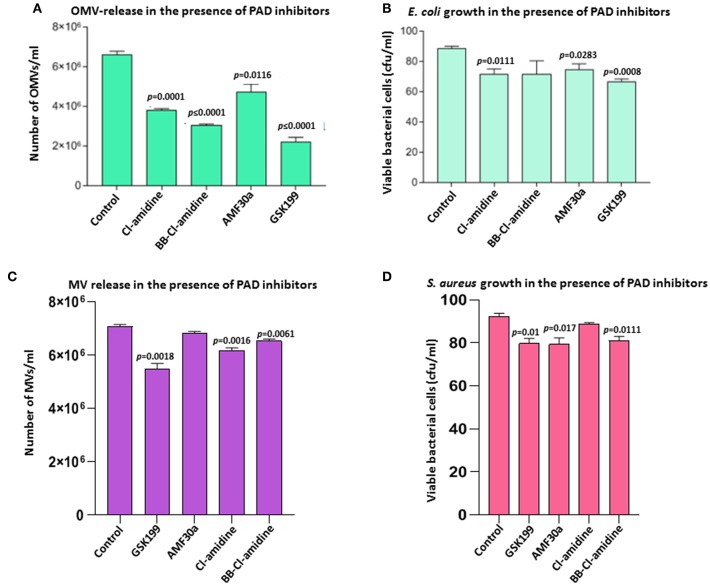
Effects of PAD inhibitors on OMV release from *E. coli* VCS257 and MV release from *S. aureus* subsp. *aureus* Rosenbach. **(A)** All PAD inhibitors showed significant OMV inhibition in *E. coli* compared to untreated controls. PAD4 inhibitor GSK199 and second generation pan-PAD inhibitor BB-Cl-amidine were the strongest inhibitors of OMV release. The PAD2-specific inhibitor AMF30a was less effective. **(B)** *E. coli* viability after 24 h PAD inhibitor treatment, represented as CFU. **(C)** PAD4 inhibitor GSK199 was the most effective MV inhibitor in *S. aureus*, reducing MV release by 22.5%. **(D)** *S. aureus* viability, represented as CFU, after 24 h treatment with PAD inhibitors is shown. The experiment was repeated thrice and the data presented are mean ± SEM of the results; exact *p*-values are indicated. Concentration of PAD-inhibitors used was as follows: PAD2 inhibitor AMF30a (5 μM), PAD4 inhibitor, GSK199 (10 μM), pan-PAD inhibitors Cl-amidine (50 μM) and BB-Cl-amidine (5 μM).

### PAD Inhibitors Inhibit OMV Release in the Gram-Negative Bacterium *E. coli* VCS257

Effects of the various PAD inhibitors on OMV release in *E. coli* are shown in Figure 2. The PAD4-specific inhibitor GSK199 showed most potent OMV inhibition with a 66.4% reduction in OMV release (*p* = 0.0001), but also most significantly affected cell viability (23.9% reduction, *p* = 0.0008). BB-Cl-amidine was the second most potent inhibitor with a 53.8% reduction in OMV release (*p* < 0.0001) and a reduction, albeit non-significant (*p* = 0.1351), of 19.3% in cell viability, as measured by CFU. Cl-amidine resulted in a 42.4% inhibition of OMV release (*p* = 0.0001) and an 18.2% decrease in cell viability (*p* = 0.0111). The PAD2-specific inhibitor AMF30a was less effective with a 28.2% reduction in OMV release (*p* = 0.0116) and caused a 14.7% reduction in cell viability (*p* = 0.0283) (Figure 2B). Furthermore, in addition to reduced total OMV numbers released after PAD inhibitor treatment, the following changes in OMV profiles were observed by NTA analysis: Cl-amidine treated *E. coli* released OMVs in the size range 20–400 nm, BB-Cl-amidine treated cells showed OMV release in the 20–500 nm size range, while AMF30a (PAD2 inhibitor) treatment resulted in an additional notable peak of OMVs at 501 nm and a second smaller peak at 701 nm. GSK199 (PAD4 inhibitor) treatment resulted in an OMV release profile of 20–400 nm, similar to that for Cl-amidine (Figures 1D–G).

### PAD Inhibitors Inhibit Membrane Vesicle (MV) Release in *S. aureus s*ubsp*. aureus* Rosenbach

The same range of PAD inhibitors as used with *E. coli* was used to examine the effect on membrane vesicle (MV) release from a Gram-positive bacterium, *S. aureus s*ubsp*. aureus* Rosenbach (Figure 2C). PAD4-specific inhibitor GSK199 resulted in the highest inhibition of MV release, with 22.5% reduction (*p* = 0.0018), while PAD2 inhibitor AMF30a showed only 3.4% reduction in OMV release (*p* = 0.0606). First generation pan-PAD inhibitor Cl-amidine resulted in a 12.5% inhibition of MV release (*p* = 0.0016), and also had the lowest negative impact on *S. aureus* cell viability (3.3%, *p* = 0.0835; Figure 2D), while second generation pan-PAD inhibitor BB-Cl-amidine showed 7.6% MV inhibition (*p* = 0.0061) (Figures 2C,D).

### Phylogenetic Reconstruction of *E. coli* and *S. aureus* PAD/AD

**Figure 3:**
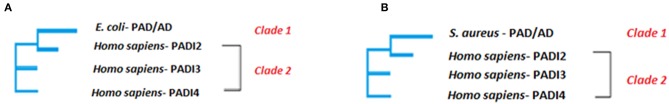
Neighbor joining tree. The phylogenetic clustering of *E. coli* **(A)** and *S. aureus* **(B)** PAD/AD, respectively is shown. The evolutionary analysis was inferred using the Neighbor-Joining method under the conditions of the Poisson distance correction model in MEGA6. Bootstrap values >50 based on 1,000 replicates are shown as nodal support. Clade 1 contains *E. coli* (GenBank: EDV68547.1) PAD/AD and Clade 2 contains human PAD2 (GenBank:NP031391), PAD3 (GenBank: AIC56498) and PAD4 (GenBank: AIC56076).

Two well supported clades were formed within the Neighbor-joining phylogeny (Figure 3) based on multiple sequence alignment of the whole amino acid sequences (using Clustal Omega), suggesting that E. coli PAD/AD (arginine deiminase RRM86073.1) is most closely related to human PAD2 (Figure 3A). The E. coli PAD/AD has a shorter, 406 amino acid (aa) sequence (arginine deiminase EDV68547.1), compared to human PAD2 (NP031391), PAD3 (AIC56498), and PAD4 (AIC56076), which are 665, 663, and 664 aa, respectively. Various single, fully conserved residues are found, while some conservation of similarity between E. coli PAD/AD with human PAD2 and PAD3 is visible that scores >0.5 in the Gonner PAM matrix (Supplementary Figure 1). For S. aureus, two well-supported clades were also formed within the Neighbor-joining phylogeny (Figure 3B), suggesting that S. aureus PAD/AD (arginine deiminase BBA25170) is also most closely related to human PAD2. The S. aureus bacterial PAD/AD has a shorter, 411 aa sequence (BBA25170), similar to that seen for E. coli PAD/AD, compared to the human PAD2, PAD3, and PAD4 isozymes, which are approximately 664 aa. Various single, fully conserved residues are found, while some conservation of similarity between S. aureus PAD/AD to human PAD2 and PAD3 is visible that scores >0.5 in the Gonner PAM matrix (Supplementary Figure 2).

### Detection of PAD/AD Protein and Citrullinated/Deiminated Protein Products in *E. coli* VCS257

**Figure 4:**
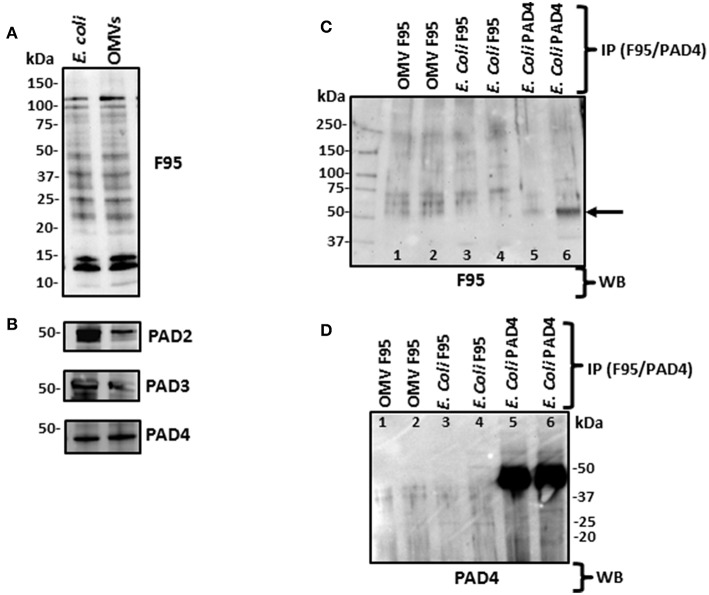
Western blotting of total deiminated proteins and PAD/AD in *E. coli* VCS257 cells and derived OMVs. **(A)** *E. coli* and derived OMVs were analyzed for total deiminated proteins (F95) as well as for cross-reactivity with human PAD2, PAD3 and PAD4. Bands in the size range of 15–120 kDa were revealed for the pan-deimination antibody (F95). **(B)** The human PAD2, 3 and 4 antibodies reacted with a band of 40–50 kDa as expected for a bacterial PAD/AD homolog. Lane 1, *E. coli* total protein extract; Lane 2, *E. coli* OMV protein extract. **(C,D)** Immunoprecipitated deiminated proteins (F95) and PAD4 bound proteins from *E. coli* VCS257 and derived OMVs. IP was performed on *E. coli* total protein and *E. coli* derived OMVs, using F95 and PAD4 antibodies, respectively. **(C)** Immunoprecipitated fractions (F95 enriched and PAD4 bound, respectively) were tested with the pan-deimination F95 antibody. A range of deiminated/citrullinated proteins was observed in *E. coli* cells and derived OMVs. Lanes 5 and 6 indicate a deiminated band at 50 kDa corresponding to *E*. coli PAD/AD (arrow) For lanes 5 and 6 the F95 enriched eluate was eluted using non-reducing and reducing elution buffer, respectively, and both F95-bound eluates obtained were thereafter run under reducing conditions in the gel. **(D)** The same fractions were tested with the PAD4 antibody. Prominent bands around the 50 kDa region (lanes 5–6) correspond to a predicted size of *E. coli* PAD/AD. Faint bands of similar sizes are also observed in the OMV F95-enriched samples (lanes 1–2) which may suggest the presence of a PAD/AD which is deiminated in both *E. coli* and derived OMVs. The protein standard is indicated in kDa on all blots. For identification of protein hits as assessed by LC-MS/MS for the F95 and PAD4 bound eluates, see Tables 1, 2.

Using Western blotting analysis, *E. coli* cell and *E. coli* OMV protein extracts were assessed for total deiminated proteins, using the anti-peptidyl-citrulline F95 antibody (MABN328, Merck, U.K.), as well as for cross-reactivity with human PAD2 (ab50257), PAD3 (ab169479), and PAD4 (ab96758) antibodies (Figure 4). For total deiminated proteins (F95), bands in the size range of 15–120 kDa were revealed in both *E. coli* cells and derived OMVs (Figure 4A). The presence of a PAD/AD-like protein was verified in *E. coli* and derived OMVs, by the detection of an expected band at 40–50 kDa, representative of a bacterial PAD/AD (Figure 4B).

### Immunoprecipitation of Deiminated Proteins From *E. coli* VCS257 and Derived OMVs

Deiminated proteins from *E. coli* and derived OMVs were immunoprecipitated using the Catch and Release® v2.0 Reversible Immunoprecipitation System (Merck, U.K.) according to the manufacturer's instructions and the anti-peptidyl-citrulline F95 antibody (MABN328, Merck) (Figures 4C,D and Supplementary Figure 3). As the PAD4 inhibitor GSK199 was here found to be the most effective OMV inhibitor (Figure 2A), immunoprecipitation using the PAD4 antibody was also carried out on *E. coli* cell protein extracts, for identification of putative PAD4 bound proteins (Figure 4 and Supplementary Figure 3). A range of F95 enriched bands was seen in both *E. coli* and OMV samples (Figure 4C). In addition, the PAD4 enriched eluate showed an F95 positive band in the expected size range of an *E. coli* PAD/AD at 45 kDa (Figure 4C, lane 6, arrow), thus indicating that *E. coli* PAD/AD itself may also be deiminated. This band was more prominent when the PAD4 antibody was used for immunoprecipitation from the *E. coli* protein lysate, showing a very strong band just below 50 kDa (Figure 4D, lanes 5–6). When testing the PAD4 bound eluate with the F95 deimination antibody, several additional positive bands were detected and may possibly represent deiminated proteins co-immunoprecipitating with the PAD4 antibody (Figure 4C; lanes 5 and 6). The F95 eluate from OMVs though also showed a reaction with the PAD4 antibody (Figure 4D; lanes 3 and 4), and therefore may indicate a deiminated PAD/AD and further deiminated proteins bound to the PAD/AD in *E. coli* derived OMVs. Such auto-deimination of the bacterial PAD/AD will though require further investigation to be fully confirmed.

### Liquid Chromatography–Mass Spectrometry (LC-MS/MS) Analysis of Deiminated Proteins From *E. coli* VCS257 and Derived OMVs

**Figure 5:**
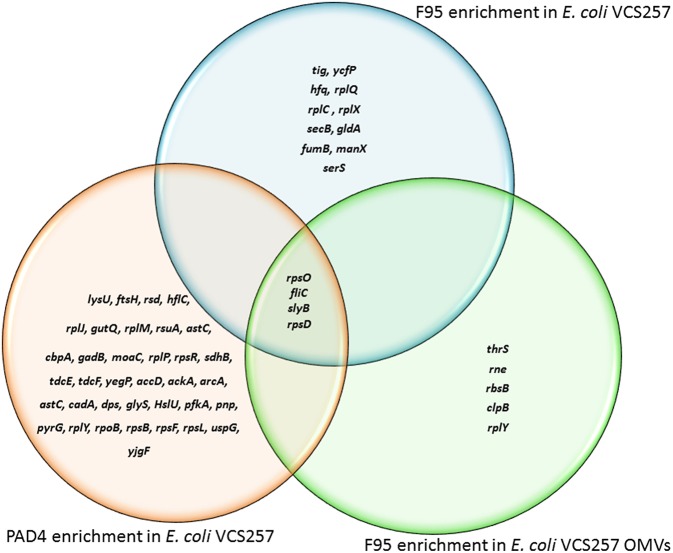
Deiminated proteins and PAD4 bound proteins identified in *E. coli* VCS257 cells and derived OMVs. The Venn diagram shows F95 enriched proteins identified from *E. coli* cells and *E. coli* OMVs, as well as PAD4 bound proteins identified from *E. coli* cells. Four proteins were common to all three eluates (for further details on protein identification see Tables 1, 2).

**Figure 6:**
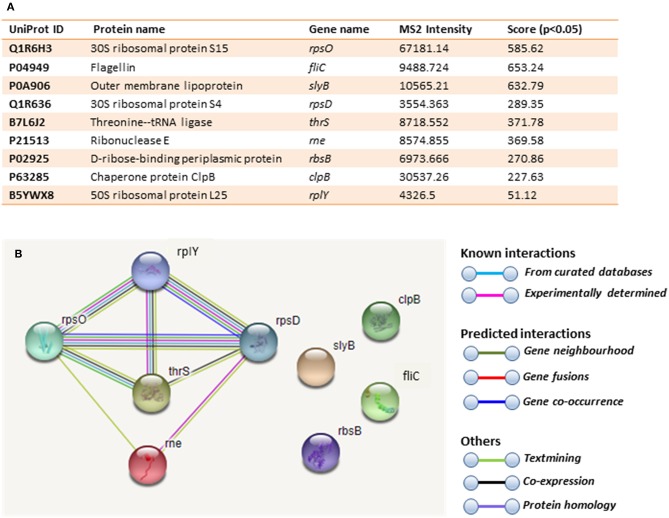
Deiminated proteins in E. coli VCS257 OMVs. Deiminated proteins were isolated by immunoprecipitation using the F95 antibody and analyzed by LC-MS/MS. **(A)**. All deiminated proteins identified in the OMV sample, with a score >50 are listed. Ions score is −10^*^Log (P), where P is the probability that the observed match is a random event. Individual ions scores >16 indicated identity or extensive homology (p<0.05). Protein scores were derived from ions scores as a non-probabilistic basis for ranking protein hits. Cut-off was set at Ions score 20. Values with 2 or more peptides per protein and a score of >50 were considered. **(B)**. String analysis of the deiminated proteins identified in E. coli OMVs. Out of 9 proteins, 5 were associated with each other, at least through text mining.

Immunoprecipitated proteins (F95 enriched and PAD4 bound) from *E. coli* cells and derived OMVs were analyzed by LC-MS/MS (Figures 5, 6 and Tables 1, **2**). Nine deiminated proteins were identified in the F95 enriched *E. coli* OMV protein sample (Figures 5, 6A). The 30s ribosomal protein S15 was unique to OMVs. As threonine-tRNA ligase (*thrS*), has not been reported in *E. coli* OMVs before, STRING (Search Tool for the Retrieval of Interacting Genes/Proteins) analysis (https://string-db.org/) was carried out to assess if *thrS* is interconnected with the other proteins in the OMV associated proteins (Figure 6B). This showed five of the nine proteins to be associated with each other, at least through text mining. Furthermore, the *E. coli* OMV-associated *thrS* is co-expressed with 30s ribosomal protein S15 (*rpsO)*, 30s ribosomal protein S4 (*rpsD*) and 50S ribosomal protein L22 (*rplY*), as well as being experimentally determined to interact with *rplY* (Figure 6B).

**Table 1:** Deiminated proteins identified by F95 enrichment in *E. coli* VCS257cells.

**UniProt ID**	**Protein name**	**Abbreviation**	**MS2 Intensity**	**Score (*p* < 0.05)^a^**
B7MQF2	Trigger factor	*tig*	81193	192.72
B7NKH1	UPF0227 protein YcfP	*ycfP*	11941	166.86
B7MSJ0	RNA-binding protein Hfq	*hfq*	256917	132.47
Q1R638	50S ribosomal protein L17	*rplQ*	126430	128.85
Q1R602	50S ribosomal protein L3	*rplC*	69461	254.77
Q1R619	50S ribosomal protein L24	*rplX*	6484.4	75.48
B7N255	Protein-export protein SecB	*secB*	136512	77.044
P14407	Fumarate hydratase class I, anaerobic	*fumB*	15233	78.27
P0A9S6	Glycerol dehydrogenase	*gldA*	220449	441.19
P69799	PTS system mannose-specific EIIAB component	*manX*	94232	56.71
B7MRV6	Serine-tRNA ligase	*serS*	16845	55.11

a Ions score is −10^*^Log(P), where P is the probability that the observed match is a random event. Individual ions scores >16 indicated identity or extensive homology (p < 0.05). Protein scores were derived from ions scores as a non-probabilistic basis for ranking protein hits. Cut-off was set at Ions score 20. Values with 2 or more peptides per protein and a score of >50 were considered.

*Deiminated proteins were isolated by immunoprecipitation using the pan-deimination F95 antibody and analyzed by LC-MS/MS. Peak files were submitted to Mascot*.

Table 1 lists proteins identified in *E. coli* cell samples that were unique in F95 enriched eluates. Table 2A lists proteins that were identified in the PAD4 enriched eluates from *E. coli* cells, indicative of PAD4 bound proteins. Table 2B summarizes common proteins identified in all three eluates (F95 enriched eluate from *E. coli* cells, F95 enriched eluate from OMVs, PAD4 enriched eluate from *E. coli* cells); proteins are shown that had two or more peptides identified and a protein score of >50.

**Table 2A:** PAD4 bound proteins identified in *E. coli* VCS257 cells.

**UniProt ID**	**Protein name**	**Abbreviation**	**MS2 Intensity**	**Score^a^ (*p* < 0.05)**
P0A895	Lysine-tRNA ligase, heat inducible	*lysU*	321997	300.12
Q8X9L0	ATP-dependent zinc metalloprotease FtsH	*ftsH*	1E+06	151.09
B7MRC1	Regulator of sigma D	*rsd*	23402	247.53
P0ABC5	Modulator of FtsH protease HflC	*hflC*	66850	189.23
Q1R5V0	50S ribosomal protein L10	*rplJ*	478659	218.99
Q8X4S5	Arabinose 5-phosphate isomerase GutQ	*gutQ*	486219	120.37
Q1R6A9	50S ribosomal protein L13	*rplM*	99365	135.62
P0AA45	Ribosomal small subunit pseudouridine synthase A	*rsuA*	50727	162.27
A7ZML6	Succinylornithine transaminase	*astC*	232079	73.58
B7MPT2	Curved DNA binding protein	*cbpA*	6123.2	90.68
P69911	Glutamate decarboxylase beta	*gadB*	387378	72.46
A7ZY39	Cyclic pyranopterin monophosphate synthase accessory protein	*moaC*	17232	67.03
Q1R613	50S ribosomal protein L16	*rplP*	475140	95.53
Q1R358	30S ribosomal protein S18	*rpsR*	312269	103.87
P07014	Succinate dehydrogenase iron-sulfur subunit	*sdhB*	48651	157.85
P42632	PFL-like enzyme TdcE	*tdcE*	29499	66.01
P0AGL3	Putative reactive intermediate deaminase TdcF	*tdcF*	161746	80.57
Q8X7I0	UPF0339 protein YegP	*yegP*	6121.7	87.63
Q0TFD0	Acetyl-coenzyme A carboxylase carboxyl transferase subunit-β	*accD*	156916	99.12
P0A6A5	Acetate kinase	*ackA*	79146	70.42
P0A9Q3	Aerobic respiration control protein ArcA	*arcA*	19035	60.23
A8A0U0	Succinylornithine transaminase	*astC*	28022	73.51
P0A9H4	Inducible lysine decarboxylase	*cadA*	716567	78.26
Q8FJM0	DNA protection during starvation protein	*dps*	51562	65.43
B7N1L1	Glycine-tRNA ligase beta subunit	*glyS*	23006	87.76
B7N2S2	ATP-dependent protease ATPase subunit HslU	*HslU*	398703	52.01
B7N2Q7	ATP-dependent 6-phosphofructokinase isozyme 1	*pfkA*	57552	62.75
B7UJ59	Polyribonucleotide nucleotidyltransferase	*Pnp*	5914.7	70.94
B7MZ76	CTP synthase	*pyrG*	17163	59.39
B6I184	50S ribosomal protein L25	*rplY*	56618	57.85
Q0TA78	DNA-directed RNA polymerase subunit beta	*rpoB*	102175	72.91
B6HZE3	30S ribosomal protein S2	*rpsB*	226035	68.17
B7NGD4	30S ribosomal protein S6	*rpsF*	151007	61.19
C4ZUJ7	30S ribosomal protein S12	*rpsL*	396281	110.55
Q8XBT3	Universal stress protein G	*uspG*	36115	159.67
P0AF94	2-iminobutanoate/2-iminopropanoate deaminase	*yjgF*	22468	105.18

a Ions score is −10^*^Log (P), where P is the probability that the observed match is a random event. Individual ions scores >16 indicated identity or extensive homology (p < 0.05). Protein scores were derived from ions scores as a non-probabilistic basis for ranking protein hits. Cut-off was set at Ions score 20. Values with 2 or more peptides per protein and a score of >50 were considered.

*Proteins were isolated by immunoprecipitation using the PAD4 antibody (ab96758) and analyzed by LC-MS/MS. Peak files were submitted to Mascot*.

**Table 2B:** Proteins identified both in F95 and PAD4 enriched samples of *E. coli* VCS257 cells and derived OMVs.

			**MS2 intensity**			
**UniProt ID**	**Protein name**	**Abbreviation**	***E. coli* F95**	***E. coli* PAD4**	**OMV F95**	**Score^a^ (*p* < 0.05)**
Q1R6H3	30S ribosomal protein S15	*rpsO*	128707	61762	67181	585.62
P04949	Flagellin	*fliC*	704599	1E+06	9488.7	653.24
P0A906	Outer membrane lipoprotein SlyB	*slyB*	289574	555800	10565	632.79
Q1R636	30S ribosomal protein S4	*rpsD*	22719	44378	3554.4	289.35

a Ions score is −10^*^Log (P), where P is the probability that the observed match is a random event. Individual ions scores >16 indicated identity or extensive homology (p < 0.05). Protein scores were derived from ions scores as a non-probabilistic basis for ranking protein hits. Cut-off was set at Ions score 20. Values with 2 or more peptides per protein and a score of >50 were considered.

*Proteins commonly identified by LC-MS/MS in both F95 and PAD4 enriched protein eluates are listed*.

### PAD Inhibitor Treatment Enhances Antibiotic Sensitivity of *E. coli* VCS257

**Figure 7:**
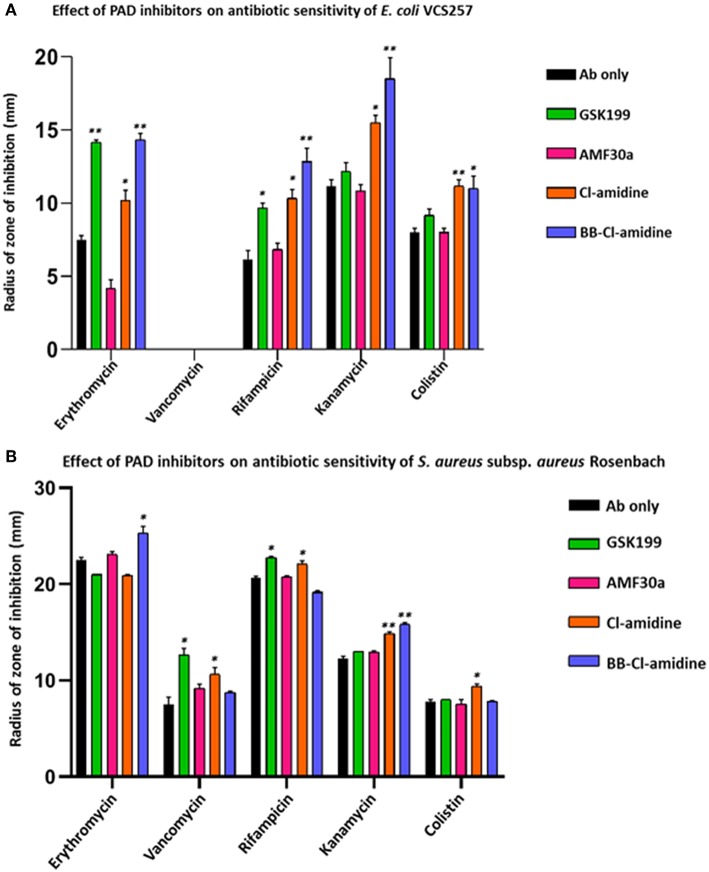
PAD inhibitors increase antibiotic sensitivity of *E. coli* VCS257 *and S. aureus* subsp*. aureus* Rosenbach. **(A)** In *E. coli*, all antibiotics (Ab), except for vancomycin, had significantly increased zones of inhibition in the presence of PAD inhibitors. BB-Cl-amidine, Cl-amidine and GSK199 were effective, increasing antibiotic sensitivity of *E. coli* to erythromycin, rifampicin, kanamycin and colistin. Vancomycin was not effective against Gram-negative bacterial colonies and did not result in a zone of inhibition in the presence of any of the PAD inhibitors. **(B)** In *S. aureus*, vancomycin, rifampicin, kanamycin and colistin significantly increased their zones of inhibition in the presence of PAD inhibitors. BB-Cl-amidine enhanced bactericidal effects of erythromycin, while vancomycin was enhanced by GSK199 and Cl-amidine. Anti-bacterial effects of rifampicin were enhanced by GSK199 and Cl-amidine. Kanamycin's zone of inhibition was increased by Cl-amidine and BB-Cl-amidine. For colistin, the zone of inhibition was increased by Cl-amidine. Concentration of PAD-inhibitors used was as follows: PAD2 inhibitor AMF30a (5 μM), PAD4 inhibitor, GSK199 (10 μM), pan-PAD inhibitors Cl-amidine (50 μM) and BB-Cl-amidine (5 μM). The experiments were carried out three times and the data presented are mean ± SEM of the results (^*^p ≤ 0.05; ^**^p ≤ 0.01).

**Figure 8:**
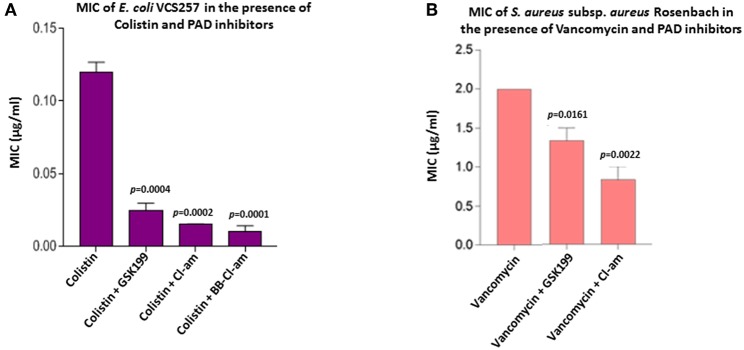
PAD inhibitors reduce the MIC value of colistin for *E. coli* VCS257 and MIC value of vancomycin for *S. aureus* subsp*. aureus* Rosenbach. **(A)** In *E. coli*, BB-Cl-amidine was most effective at lowering the MIC value of colistin, reducing MIC by 91.6%. Cl-amidine decreased MIC by 87.4%, being the second most effective OMV-inhibitor lowering the MIC value of colistin. GSK199 lowered MIC by 76%. The experiment was repeated thrice and the data presented are mean ± SEM of the results; exact *p*-values are indicated. **(B)** In *S. aureus*, Cl-amidine increased the effectiveness of vancomycin by at least 62.5%, while GSK199 resulted in a 25% decrease in MIC. The experiments were repeated thrice and the data presented are mean ± SEM of the results; exact *p*-values are shown.

Kanamycin was the most effective antibiotic against *E. coli* in the absence of PAD inhibitors (Figure 7A). In the presence of PAD inhibitors, GSK199 most significantly enhanced the effects of erythromycin, measured as a percentage increase in the zone of inhibition on the lawn of *E. coli*, by 88.9% (*p* = 0.0025); that of rifampicin by 56.7% (*p* = 0.0561) and that of colistin by 14.6% (*p* = 0.2495). BB-Cl-amidine was most effective when used in combination with rifampicin, increasing the zone of inhibition by 106.45% (*p* = 0.0025) and that of kanamycin by 65.6% (*p* = 0.0186). Cl-amidine caused enhancement of antibiotic sensitivity in combination with rifampicin (43.8%; *p* = 0.045), erythromycin (35.6%; *p* = 0.0572), and kanamycin (38.8%; *p* = 0.0390), compared to antibiotic alone (Figure 8). AMF30a was not effective in sensitizing *E. coli* to any of the antibiotics tested (see Supplementary Figure 4A, showing the agar plates). Importantly, there were no zones of inhibition seen in the agar plates which were only treated with the inhibitor discs, thus ruling out any inhibition on bacterial growth in the absence of antibiotics (Supplementary Figure 4B).

### PAD Inhibitors Enhance Antibiotic Sensitivity of *S. aureus* subsp. *aureus* Rosenbach

PAD inhibitors significantly enhanced antibiotic effectivity against *S. aureus* (Figure 7B)**.** For erythromycin, BB-Cl-amidine significantly enhanced antibacterial effects, by 18.2% (*p* = 0.0234). For vancomycin, GSK199 was most effective, with a 69.3% increase in antibiotic effectivity (*p* = 0.0250), while Cl-amidine was also significant (42.7%, *p* = 0.0354). (Figure 7B). For rifampicin, GSK199 and Cl-amidine significantly increased the zone of inhibition (10.1%; *p* = 0.0202) and 6.4%; *p* = 0.0239, respectively). The antibacterial effects of kanamycin were also significantly increased by both Cl-amidine and BB-Cl-amidine by 20.8% (*p* = 0.0055) and 28.9%; (*p* = 0.0101), respectively. For colistin, Cl-amidine increased the zone of inhibition by 21.5% (*p* = 0.0444). There were no zones of inhibition seen in the agar plates treated with the PAD inhibitor discs alone, indicating no effect on bacterial growth (Supplementary Figures 4C,D).

### PAD Inhibitors Decrease MIC Value of Colistin Against *E. coli* VCS257

As the minimum inhibitory concentration (MIC) value of colistin against Gram-negative bacteria has been inconsistently reported in the literature it was further investigated here (Figure 8A). BB-Cl-amidine and Cl-amidine resulted in 91.6% (*p* = 0.0001) and 87.4% (*p* = 0.0002) reduction in MIC, respectively, while GSK199 lowered the MIC by 76% (*p* = 0.0004) (Figure 8A). There were no zones of inhibition seen in the plates which were only treated with the inhibitor discs, indicating no bactericidal effect due to inhibitors alone.

### PAD Inhibitors Decrease MIC Value of Vancomycin Against *S. aureus* subsp. *aureus* Rosenbach

The MIC value of vancomycin against *S. aureus* has been inconsistently reported in the literature and was thus investigated further. Figure 8B shows the effect of the most effective MV-inhibiting PAD inhibitors on the MIC of vancomycin; Cl-amidine showed a 62.5% reduction (*p* = 0.0022) while GSK199 lowered MIC by 25% (*p* = 0.0161) (Figure 8B). There was no effect on MIC of colistin in the presence of inhibitors alone.

## Discussion

For analysis of bacterial OMV/MVs in this study, isolation and quantification approaches using ultracentrifugation and NTA analysis were used, similar as performed by other groups (McCaig et al., 2013; Klimentova and Stulik, 2015; Roier et al., 2016). OMVs have previously been reported to fall mainly in the size range 10–300 nm (Kulkarni et al., 2015; Huang et al., 2016), and similar profiles were observed here. OMVs were also further characterized morphologically using TEM, and by Western blotting analysis using the outer membrane specific marker, OmpC (Figure 1). Furthermore, some change in OMV profile was observed in response to PAD inhibitor treatment, and this varied between inhibitors used, showing a change in shift of vesicle size populations after treatment with the different PAD inhibitors.

Here, an *E. coli* VCS257 citrullinome was identified for the first time, using F95 enrichment and LC-MS/MS analysis, confirming the presence of deiminated/citrullinated proteins in *E. coli* cells and *E. coli* derived OMVs. In bacteria, studies on PAD/AD homologs have been limited and hitherto mainly reported in *P. gingivalis* (Mangat et al., 2010; Bielecka et al., 2014); a *Gingivalis* citrullinome has also been described (Stobernack et al., 2016). An *E. coli* PAD/AD-like protein was detected here at approximatly 40–50 kDa, similar to that found in *P. gingivalis* (Bielecka et al., 2014; Gabarrini et al., 2018), while in comparison human PADs are 72–75 kDa (Vossenaar et al., 2003; Kosgodage et al., 2018). Multiple sequence analysis of *E. coli* and *S. aureus* suggested that PAD/AD of *E. coli* and *S. aureus* are most closely related to human PAD2.

Interestingly, for inhibiting OMV/MV release from Gram-negative and Gram-positive bacteria the PAD4-specific inhibitor GSK199 was most effective while the PAD2-specific inhibitor AMF30a was least effective. Therefore, it may be postulated that the tertiary conformation of both *E. coli* and *S. aureus* PAD/ADs may be more similar to human PAD4, although the amino acid sequence alignment analysis indicates more similarity to PAD2, and this will require further investigation. In *P. gingivalis*, also a Gram-negative bacterium, PAD is believed to be evolutionarily only remotely related to human PAD2 despite the fact that both catalyze the same chemical reaction (Rodríguez et al., 2009; Bereta et al., 2019) and furthermore, point-mutation variants with differing deimination activity have also been reported in *Gingivalis* (Gabarrini et al., 2018; Bereta et al., 2019).

Here, F95 enrichment analysis revealed a range of deiminated proteins, both in *E. coli* cells and their derived OMVs, indicating lateral transfer of deiminated proteins via OMVs. Besides roles in bacterial communication, this may possibly also affect host-pathogen interactions, including immune evasion via modification of the host's proteins. For example, citrullination/deimination of complement component C5a, upon treatment with *P. gingivalis* OMVs, as opposed to *in vitro* treatment of C5a with PAD, has been shown to contribute to bacterial immune evasion by decreasing the chemotactic ability of neutrophils (Bielecka et al., 2014).

The citrullinome of *E. coli* VCS257 observed here, revealed indeed a range of metabolic, stress-response related and membrane proteins, therefore indicating diverse roles for protein deimination in bacterial cell function. Deiminated proteins identified in *E. coli* derived OMVs included threonine-tRNA ligase, which has not been described as being deiminated in bacterial OMVs before, but has been shown to be in OMVs from *Streptococcus suis* (Haas and Grenier, 2015), albeit not in deiminated form. Threonine-tRNA ligase, also known as threonine-tRNA synthetase, belongs to the family of aminoacyl-tRNA synthetases, which are involved in RNA splicing and transcriptional and translational regulation. They link amino acids to their cognate transfer RNAs (tRNA) in aminoacylation reactions, that establish the connection between a specific amino acid and a nucleotide triplet anticodon embedded in the tRNA (Schimmel, 2008). Also, 30S ribosomal protein was identified as being deiminated in OMVs. It is one of the primary rRNA binding proteins that bind directly to 16S rRNA, where it helps nucleate assembly of the platform of the 30S subunit by binding and bridging several RNA helices of the 16S rRNA (Smith et al., 2018). In addition, 30S ribosomal protein S4 (*rpsD*) and S15 (*rpsO*) were identified as deiminated both in the *E. coli* cells as well as their derived OMVs. Previously, 40S ribosomal protein has been reported as a substrate of PAD4 mediated deimination/citrullination in HEK 293Tcells (Guo et al., 2011).

Furthermore, evidence of a PAD/AD-like protein exported in *E. coli* VCS257 derived OMVs was found. When probing the PAD4 enriched eluate with the F95 antibody, a faint positive band in the expected range for a putative bacterial PAD/AD, in the region of 50 kDa region was observed, indicating that possibly the *E. coli* PAD/AD may be deiminated itself, although the LC MS-MS analysis did not reveal a PAD/AD-like protein hit. Such a possibility of PAD/AD auto-deimination would though align with previous studies on auto-deimination of mammalian PAD4 (Slack et al., 2011) but will require further in-depth investigation. In a recent study on OMVs derived from *P. gingivalis*, it was found that *Gingivalis* PAD was abundant in OMVs, although no assessment was made of a deiminated PAD form or of deiminated proteins exported in OMVs (Gabarrini et al., 2018).

The effect of PAD inhibitors on OMV/MV release, as previously established for EV release in eukaryotic cells (Kholia et al., 2015; Kosgodage et al., 2017, 2018; Gavinho et al., 2019), reveals a phylogenetically conserved pathway from bacteria to mammals. This is also in line with findings that many commonly expressed proteins including chaperone proteins, ribonuclease, outer membrane lipoprotein, 50S ribosomal protein L22 and flagellin are believed to be targets of protein deimination (Huang et al., 2016; Claushuis et al., 2018). Importantly, the present study shows that PAD inhibitors can be used to enhance antibiotic sensitivity of selected antibiotics. PAD4 inhibitor GSK199, alongside the pan-PAD inhibitors were effective for OMV/MV inhibition and sensitization to antibiotic treatment. Differences in mechanisms of action for the selected PAD inhibitors used in this study may need to be considered, both with regards to inhibiting vesicle release and the synergism observed with each antibiotic. Interestingly, while PAD4-inhibitor GSK199 was overall the strongest OMV/MV inhibitor, albeit the pan-PAD inhibitors showed a similar trend, in some cases the pan-PAD inhibitors (Cl-am and BB-Cl-am) were more effective in sensitization to antibiotic treatment. It must also be taken into consideration that BB-Cl-amidine and Cl-amidine are hydrophilic, while AMF30a and GSK199 are hydrophobic compounds. Furthermore, GSK199 is highly lipophilic which may facilitate its uptake in the cell. The difference in hydrophobicity of the PAD inhibitors could have played a role in cell penetration, in addition to differing in specificity for inhibition of the bacterial PAD/AD, and this may therefore also have affected OMV/MV release. A recent review has demonstrated the presence of different types of vesicles released from both Gram-negative and Gram-positive bacteria, indicating also that the composition of the cell membrane plays a role in vesiculation (Toyofuku et al., 2019). The presence of a thickened peptidoglycan cell wall in Gram-positive bacteria restricts the penetration of most drugs into the cells, which suggests the need of an alternative receptor-mediated transport system (Liu et al., 2018). However, the high lipid content of the Gram-negative cell membrane with a thin layer of peptidoglycan increases membrane fluidity thus facilitating OMV release (Roier et al., 2016). This may also facilitate the bacterial cell wall penetration of lipid soluble drugs and elicit a more effective response, as indeed observed here for GSK199.

While PAD4-mediated neutrophil extracellular trap (NET) formation is a well-known bactericidal and anti-pathogenic mechanism of the immune system (Li et al., 2010; Claushuis et al., 2018; Magnadóttir et al., 2018), we have now revealed here another antibacterial mechanism, namely via PAD/AD-mediated inhibition of bacterial OMV/MV release. Bacteria may indeed utilize their PAD/AD in several ways for modulation of the host immune system and immune invasion, including via the generation of neo-epitopes, modification of host's immune proteins, such as C5a, and also through the release of OMV/MVs. While many studies on OMVs have been based on Gram-negative bacterial species (Pérez-Cruz et al., 2013; Bonnington and Kuehn, 2016; Roier et al., 2016), MV release from *S. aureus* has also been shown by different groups (Lee et al., 2009; Gurung et al., 2011). Interestingly, MV secretion and improper vancomycin treatment have been correlated with biofilm formation by methicillin-resistant *Staphylococcus aureus* (MRSA) (He et al., 2017).

When assessing the effectivity of PAD inhibition to enhance susceptibility of Gram-positive and Gram-negative bacterial species to antibiotic treatment, *E. coli* VCS257 were rendered more sensitive to erythromycin, rifampicin, kanamycin and colistin, while *S. aureus* subsp. *aureus* Rosenbach became more sensitive to all five antibiotics tested. This increased sensitivity in both bacterial species varied though somewhat, depending on the PAD-specific inhibitors used. It must also be noted that while the zone of inhibition was statistically significant in *S. aureus*, the proportional differences observed in antibiotic sensitization were not as high as those observed in some cases for *E. coli* and therefore it will require further investigation how physiologically significant such lower, albeit statistically significant, effects are. It can also be postulated that bacteria may use PAD/AD mediated deimination as a mode of a hitherto non-described microbial strategy to evade antibiotic action, and this might also explain some differences observed with the different PAD inhibitors and will require further in-depth investigation. There was no antibacterial effect of vancomycin on *E. coli* VCS257, confirming its limited effectiveness on Gram-negative species and the previously established resistance of *E. coli* to vancomycin, due to its inability to significantly penetrate the outer membrane (Zhou et al., 2015). Here, colistin and vancomycin effectivity was found to be enhanced in the presence of PAD inhibitors in *E. coli* and *S. aureus* respectively, decreasing MIC value at varying degrees. This indicates that lower concentrations of the antibiotic may be used to treat infections with minimal damage to healthy cells. It has previously been shown that the presence of calcium decreases the bactericidal effect of colistin on *Paenibacillus polymyxa*, which suggests that Ca^2+^ modulates a protective barrier against colistin (Yu et al., 2015). As the PADs are calcium-activated enzymes, it can be postulated that downstream PAD/AD activation and resulting OMV/MV release via a PAD/AD pathway may be a measure of bacterial defense against colistin treatment.

Our current study is the first to describe effects of PAD inhibitors on OMV/MV release in both a Gram-positive and Gram-negative bacterial species. PAD/AD inhibition approaches in bacteria have previously been discussed in relation to oral *Gingivalis* and the association to initiation of autoimmunity via the generation of neo-epitopes (Mangat et al., 2010; Montgomery et al., 2016). Pharmacological inhibition of PADs, using Cl-amidine or a PAD2/PAD4 inhibitor has also been shown to improve survival in several murine models of sepsis and LPS-induced endotoxemia (Zhao et al., 2016; Biron et al., 2017; Liang et al., 2018). Here we have shown that PAD inhibition had a significant effect on antibiotic sensitization in both species, albeit to a lower extent in *S. aureus*. Therefore, further identification and assessment of candidate OMV/MV inhibitors may allow for tailored application according to bacteria type and specific antibiotics.

Previous studies have discussed the use of OMVs for example as drug delivery vehicles (Ellis and Kuehn, 2010; Gujrati et al., 2014; Gerritzen et al., 2017; Jain and Pillai, 2017; Jan, 2017; Wang et al., 2018). OMVs have also been tested as delivery vehicles for targeted gene silencing using siRNA-packaged OMVs (Alves et al., 2016), although the exact mechanism for packaging proteins and other reagents in OMVs still remains to be fully understood. There is also an increasing interest in identification of OMV sub-populations (Pérez-Cruz et al., 2016; Bonnington and Kuehn, 2017; Turner et al., 2018; Cooke et al., 2019; Toyofuku et al., 2019; Zavan et al., 2019), as well as in assessing the importance of OMV size for cellular uptake and entry (Turner et al., 2018). Therefore, changes observed here in the NTA spectra of OMVs in response to PAD inhibitor treatment (Figures 1D–G) may be of some interest in addition to the observed reduction in total amounts of OMV/MVs released.

For the first time, the potential of using PAD inhibitors to enhance antibiotic sensitivity has been assessed, in both a Gram-positive and Gram-negative bacterial species, revealing a phylogenetically conserved PAD/AD pathway of membrane vesicle release. This may open avenues for tailored OMV/MV inhibition in combination with selected antibiotics, according to bacterial type, to regulate biofilm formation and tackle antibiotic resistance.

## Conclusion

This study reveals a phylogenetically conserved PAD/AD pathway of OMV/MV release in bacteria that can be pharmacologically modulated to sensitize bacteria to antibiotic treatment. For the first time, a citrullinome of *E. coli* VCS257 and associated OMVs is described, indicating lateral transfer of deiminated proteins via OMVs. Our findings highlight new applications for PAD inhibitors to regulate OMV/MV release and to enhance antibiotic sensitivity in both Gram-positive and Gram-negative bacteria.

## Data Availability

The raw data supporting the conclusions of this manuscript will be made available by the authors, without undue reservation, to any qualified researcher.

## Author Contributions

UK, PM, GM, IK, DB, BA, and SL performed the experiments. UK, SL, and JI analyzed the data. PM, GM, IK, AN, SL, and JI provided resources. UK, SL, and JI designed the study and wrote the manuscript. All authors critically reviewed the manuscript.

### Conflict of Interest Statement

The authors declare that the research was conducted in the absence of any commercial or financial relationships that could be construed as a potential conflict of interest.

## Supplementary Material

Supplementary Figure 1 Multiple sequence alignment of *E. coli* AD and human PADs. The evolutionary relationship between *E. coli* AD and human PAD2, 3 and 4 is shown; (^*^) indicates positions which have a single, fully conserved residue; (:) indicates conservation between groups of strongly similar properties - scoring >0.5 in the Gonnet PAM 250 matrix; (.) indicates conservation between groups of weakly similar properties, scoring <0.5 in the Gonnet PAM 250 matrix.

Supplementary Figure 2 Multiple sequence alignment of *S. aureus* AD and human PADs. The evolutionary relationship between *S. aureus* AD and human PAD2, 3 and 4 is shown; (^*^) indicates positions which have a single, fully conserved residue; (:) indicates conservation between groups of strongly similar properties—scoring >0.5 in the Gonnet PAM 250 matrix; (.) indicates conservation between groups of weakly similar properties, - scoring <0.5 in the Gonnet PAM 250 matrix.

Supplementary Figure 3 Western blotting of immunoprecipitated deiminated proteins (F95) and PAD4 bound proteins from *E. coli* VCS257 and derived OMVs. **(A)** Same figure as 4C, but also showing unbound fractions (flow-through) from OMVs and *E. coli* after IP, using the F95 antibody. **(B)** Same figure as 4D, but also showing unbound fractions (flow-through) from OMVs and *E. coli* after IP, using the PAD4 antibody.

Supplementary Figure 4 Agar plates showing the Kirby-Bauer disk diffusion test for *E. coli* VCS257 and *S. aureus* subsp. *aureus* Rosenbach. **(A)** Disk diffusion test for *E. coli* VCS257. **(B)** Effects of PAD inhibitor disks alone on *E. coli* are shown. **(C)** Disk diffusion test for *S. aureus* subsp. *aureus* Rosenbach. **(D)** Effects of PAD inhibitors alone on *S. aureus* are shown. Disks containing the following antibiotics are indicated: erythromycin **(E)**, vancomycin (V), Rifampicin (R), kanamycin (K), colistin (C). PAD inhibitors used were GSK199 (10 μM; PAD4 inhibitor), Cl-amidine (50 μM; pan-PAD inhibitor), BB-Cl-amidine (5 μM; pan-PAD inhibitor), AMF30a (5 μM; PAD2 inhibitor).
