# Supplementary material for: Peptidylarginine Deiminase Inhibitors Reduce Bacterial Membrane Vesicle Release and Sensitize Bacteria to Antibiotic Treatment
Source: Front Cell Infect Microbiol. 2019 Jun 27;9:227. doi: 10.3389/fcimb.2019.00227 (PMC6610471; doi:10.3389/fcimb.2019.00227)
Supplement: Supplementary Figure 1 — Multiple sequence alignment of E. coli AD and human PADs. The evolutionary relationship between E. coli AD and human PAD2, 3 and 4 is shown; (*) indicates positions which have a single, fully conserved residue; (:) indicates conservation between groups of strongly similar properties - scoring >0.5 in the Gonnet PAM 250 matrix; (.) indicates conservation between groups of weakly similar properties, scoring <0.5 in the Gonnet PAM 250 matrix. [file Table_1.DOCX]

**Supp Fig 1.** **Multiple sequence alignment of *E. coli* AD and human PADs.**

*E.coli* ------------------------------------------------------------ 0

PADI2 mlrertvrlqygsrveavyvlgtylwtdvysaapagaqtfslkhsehvwvevvrdgeaee 60

PADI3 mslqrivrvslehptsavcvagvetlvdiygsvpegtemfevygtpgvdiyispnmerg- 59

PADI4 maqgtlirvtpeqpthavcvlgtltqldicssapedctsfsinaspgvvvdiahsppak- 59

*E.coli* ------------------------------------------------------------ 0

PADI2 vatngkqrwllspsttlrvtmsqasteassdkvtvnyydeegsipidqaglfltaieisl 120

PADI3 reradtrrwrfdatleiivvmnspsndlndshvqisyhssheplplayavlyltcvdisl 119

PADI4 kkstgsstwpldpgvevtltmkaasgstgdqkvqisyygpktp--pvkallyltaveisl 117

*E.coli* ------------------------------------------------------------ 0

PADI2 dvdadrdgvve--knnpkkaswtwgpegqgaillvncdretpwlpkedcrdekvyskedl 178

PADI3 dcdlncegr--qdrnfvdkrqwvwgpsgyggillvncdrddpscdvqdncdqhvhclqdl 177

PADI4 caditrtgkvkptravkdqrtwtwgpcgqgaillvncdrdnlessamdceddevldsedl 177

*E.coli* ---------------------mekhyvgseigqlrsvmlhrpnlslkrltpsncqellfd 39

PADI2 kdmsqmilrtkgpdrlpagyeivlyismsdsdkvgvfyvenpff------gqryihilgr 232

PADI3 edmsvmvlrtqgpaalfddhklvlhtssydakraqvfhicgpedv-----ceayrhvlgq 232

PADI4 qdmslmtlstktpkdfftnhtlvlhvarsemdkvrvfqatrgkl------sskcsvvlgp 231

: : : : . . :*

*E.coli* ----dvlsverageehdifantlrq-----qgievllltdlltqtldipeakswlletqi 90

PADI2 rklyhvvkytggsaellffveglcfpdegfsglvsihvs---------------lleyma 277

PADI3 dkv-syevprlhgdeerffveglsfpdagftglisfhvt---------------llddsn 276

PADI4 kwpshylmvpggkhnmdfyvealafpdtdfpglitltis---------------lldtsn 276

: ::.: * *: : :: **:

*E.coli* sdyrlgptfatd----vrtwlaemshrdlarhlsggltyseipasiknmvvdthdindfi 146

PADI2 qdipltpiftdtvifriapwimtpnilppvsv---------fvccmkdnylflkevknlv 328

PADI3 edfsaspiftdtvvfrvapwimtpstlpplev---------yvcrvrnntcfvdavaela 327

PADI4 lelpeavvfqdsvvfrvapwimtpntqppqev---------yacsifenedflksvttla 327

: * : *: . . : : . : :

*E.coli* mkplp----nhlftrdtscwiyngvsinpmakparq----------retnnlra------ 186

PADI2 ektncelkvcfqylnrgdrwiqdeiefgyieaphkgfpvvldsprdgnlkdfpvkellgp 388

PADI3 rkagcklticpqaenrndrwiqdemelgyvqaphktlpvvfdsprngelqdfpykrilgp 387

PADI4 mkakcklticpeeenmddqwmqdemeigyiqaphktlpvvfdsprnrglkefpikrvmgp 387

* . . *: : :.:. : * : :::

*E.coli* ---iyrwhpqfaggefikyfgdeninydhatleggdvlvigrgavligmserttpqgief 243

PADI2 dfgyvtreplfesvtsldsfgnlevs--ppvtvngktyp--lgriligss-fplsggrr- 442

PADI3 dfgyvtreprdrsvsgldsfgnlevs--ppvvangkeyp--lgriliggn-lpgssgrr- 441

PADI4 dfgyvtrgpqtggisgldsfgnlevs--ppvtvrgkeyp--lgrilfgdscypsndsrq- 442

* . :. **: ::. . *. * :*:* . . .

*E.coli* laqal--fkhrqaeqviavelpkhrscmhldtvmthididtfsvypevvrpdvncwtltp 301

PADI2 mtkvvrdflkaqqvqapvelysdwltvghvdefmsfvpipgtkkflllmastsacyklfr 502

PADI3 vtqvvrdflhaqkvqppvelfvdwlavghvdeflsfvpapdgkgfrmllaspgacfklfq 501

PADI4 mhqalqdflsaqqvqapvklysdwlsvghvdeflsfvpapdrkgfrlllasprscyklfq 502

: :.: * * * . . : *:* .::.: . : :: *:.*

*E.coli* ----dghgglkrtqestllhaie-----kalgidqvrli--ttggdafeaereqwndann 350

PADI2 ekqkdghgeai------mfkglggms-skritinkilsneslvqenlyfqrcldwnrdil 555

PADI3 ekqkcghgral------lfqgvvddeqvktisinqvlsnkdlinynkfvqscidwnrevl 555

PADI4 eqqneghgeal------lfegikkkkqq---kiknilsnktlrehnsfvercidwnrell 553

*** ::..: *.:: : : :**

*E.coli* v--ltlrpgvvvgyerniwtnekydkag-----itvlpipgdelg------rgrggarcm 397

PADI2 kkelglteqdiidlp-alfkmdedhraraffpnmvnmivldkdlgipkpfgpqveeeccl 614

PADI3 krelglaecdiidip-qlfkte-rkkataffpdlvnmlvlgkhlgipkpfgpiingcccl 613

PADI4 krelglaesdiidip-qlfklkefskaeaffpnmvnmlvlgkhlgipkpfgpvingrccl 612

* * ::. ::. . :* :. : : ...** *:

*E.coli* scpl----hrdgi-------------------------------------- 406

PADI2 emhvrglleplglectfiddisayhkflgevhcgtnvrrkpftfkwwhmvp 665

PADI3 eekvrslleplglhctfiddftpyhmlhgevhcgtnvcrkpfsfkwwnmvp 664

PADI4 eekvcslleplglqctfindfftyhirhgevhcgtnvrrkpfsfkwwnmvp 663

. : . *:

**Supp Fig 2. Multiple sequence alignment of *E. coli* AD and human PADs.**

*S.aureus* ------------------------------------------------------------ 0

PADI2 mlrertvrlqygsrveavyvlgtylwtdvysaapagaqtfslkhsehvwvevvrdgeaee 60

PADI3 mslqrivrvslehptsavcvagvetlvdiygsvpegtemfevygtpgvdiyispnmerg- 59

PADI4 maqgtlirvtpeqpthavcvlgtltqldicssapedctsfsinaspgvvvdiahsppak- 59

*S.aureus* ------------------------------------------------------------ 0

PADI2 vatngkqrwllspsttlrvtmsqasteassdkvtvnyydeegsipidqaglfltaieisl 120

PADI3 reradtrrwrfdatleiivvmnspsndlndshvqisyhssheplplayavlyltcvdisl 119

PADI4 kkstgsstwpldpgvevtltmkaasgstgdqkvqisyygpktp--pvkallyltaveisl 117

*S.aureus* ------------------------------------------------------------ 0

PADI2 dvdadrdgvve--knnpkkaswtwgpegqgaillvncdretpwlpkedcrdekvyskedl 178

PADI3 dcdlncegr--qdrnfvdkrqwvwgpsgyggillvncdrddpscdvqdncdqhvhclqdl 177

PADI4 caditrtgkvkptravkdqrtwtwgpcgqgaillvncdrdnlessamdceddevldsedl 177

*S.aureus* --------mtdgpikvnse---------------------------------igalkt-v 18

PADI2 kdmsqmilrtkgpdrlpagyeivlyismsdsdkvgvfyvenpff-gqryihilgrrklyh 237

PADI3 edmsvmvlrtqgpaalfddhklvlhtssydakraqvfhicgpedvceayrhvlgqdkv-s 236

PADI4 qdmslmtlstktpkdfftnhtlvlhvarsemdkvrvfqatrgkl-sskcsvvlgpkwpsh 236

*. * . :*

*S.aureus* llkrpgkelenlvpdyldgllfddipylevaqkehdhfaqvlreegvevlyleklaaesi 78

PADI2 vvkytggs--aellffveglcfpdegfsglvsih--------------vslleymaqdi- 280

PADI3 yevprlhg--deerffveglsfpdagftglisfh--------------vtllddsnedf- 279

PADI4 ylmvpggk--hnmdfyvealafpdtdfpglitlt--------------islldtsnlel- 279

:::.* * * : : : *: :

*S.aureus* enpqvrsefiddvlaeskktilgheeeikalfatlsnqelvdkimsgvrkeeinpkcthl 138

PADI2 ---pltpiftdtvifriapwim---------------------------tpnilppvsvf 310

PADI3 ---saspiftdtvvfrvapwim---------------------------tpstlpplevy 309

PADI4 ---peavvfqdsvvfrvapwim---------------------------tpntqppqevy 309

* * *: . *: . . *

*S.aureus* veymddkypfyldpmpnlyftrdpqasighgitinrmfwrarrresifiqyivkhhprf- 197

PADI2 vccmkdnylf-lkevknlvektncelkvcfqylnrgdrwiqdeie---fgyieaphkgfp 366

PADI3 vcrvrnntcf-vdavaelarkagcklticpqaenrndrwiqdeme---lgyvqaphktlp 365

PADI4 acsifenedf-lksvttlamkakcklticpeeenmddqwmqdeme---igyiqaphktlp 365

. : :: * :. : * . : .: * . * : *: * :

*S.aureus* -----------kdanipiwldrdcpf------------------------nieggdelvl 222

PADI2 vvldsprdgnlkdfpvkellgpdfgyvtreplfesvtsldsfgnlevsppvtvngktypl 426

PADI3 vvfdsprngelqdfpykrilgpdfgyvtreprdrsvsgldsfgnlevsppvvangkeypl 425

PADI4 vvfdsprnrglkefpikrvmgpdfgyvtrgpqtggisgldsfgnlevsppvtvrgkeypl 425

:: :. * : *. *

*S.aureus* skevlaigvsertsaqaieklarrif-----enpqatfkkvva----------ieiptsr 267

PADI2 griligss-fplsggrrmtkvvrdflkaqqvqapvelysdwltvghvdefmsfvpipgtk 485

PADI3 griliggn-lpgssgrrvtqvvrdflhaqkvqppvelfvdwlavghvdeflsfvpapdgk 484

PADI4 grilfgdscypsndsrqmhqalqdflsaqqvqapvklysdwlsvghvdeflsfvpapdrk 485

.: ::. . ...: : : : :: : * : . :: : * :

*S.aureus* tfmhld----tvftmidydkftmhsailkaeg----------------------nmnifi 301

PADI2 kflllmastsacyklfrekqkdghgeaimfkglggms-skritinkilsneslvqenlyf 544

PADI3 gfrmllaspgacfklfqekqkcghgrallfqgvvddeqvktisinqvlsnkdlinynkfv 544

PADI4 gfrlllasprscyklfqeqqneghgeallfegikkkkqq---kiknilsnktlrehnsfv 542

* * : :.:: .: *. : :* : * :.

*S.aureus* ieyddvnkdiaikqssh----------------------lkdtledvlgiddiqfiptgn 339

PADI2 qrcldwnrdilkkelglteqdiidlpalfkmdedhraraffpnmvnmivldkdlgipkpf 604

PADI3 qscidwnrevlkrelglaecdiidipqlfkte-rkkataffpdlvnmlvlgkhlgipkpf 603

PADI4 ercidwnrellkrelglaesdiidipqlfklkefskaeaffpnmvnmlvlgkhlgipkpf 602

* *::: :: . : : ::: :.. **.

*S.aureus* gdvidgareqwndgsntlcirpgvvvtydrnyvsndllrqkgikvieisgselvr-grgg 398

PADI2 gpqveeec----------clemhv----------rglleplglectfiddisayhkflge 644

PADI3 gpiingcc----------cleekv----------rslleplglhctfiddftpyhmlhge 643

PADI4 gpvingrc----------cleekv----------cslleplglqctfindfftyhirhge 642

* :: *:. * .**. *:. *.. : *

*S.aureus* prcmsqplfredi--------- 411

PADI2 vhcgt-nvrrkpftfkwwhmvp 665

PADI3 vhcgt-nvcrkpfsfkwwnmvp 664

PADI4 vhcgt-nvrrkpfsfkwwnmvp 663

:* : : *: :
