# Supplementary figures and images for: Peptidylarginine Deiminase Inhibitors Reduce Bacterial Membrane Vesicle Release and Sensitize Bacteria to Antibiotic Treatment
Source: Front Cell Infect Microbiol. 2019 Jun 27;9:227. doi: 10.3389/fcimb.2019.00227 (PMC6610471; doi:10.3389/fcimb.2019.00227)

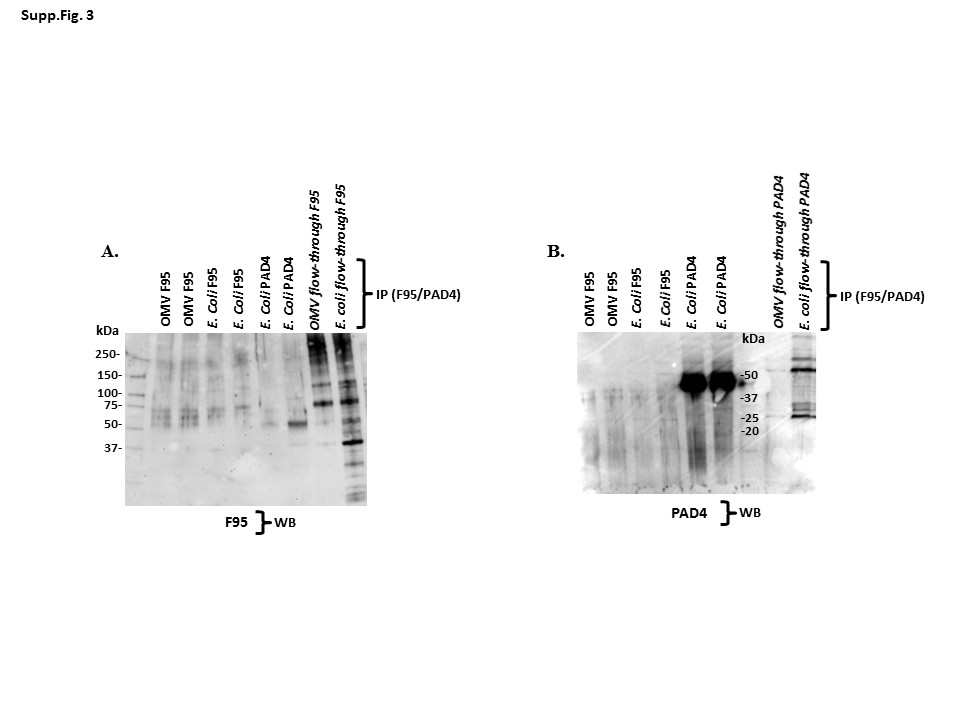

Supplement: Supplementary Figure 3 — Western blotting of immunoprecipitated deiminated proteins (F95) and PAD4 bound proteins from E. coli VCS257 and derived OMVs. (A) Same figure as 4C, but also showing unbound fractions (flow-through) from OMVs and E. coli after IP, using the F95 antibody. (B) Same figure as 4D, but also showing unbound fractions (flow-through) from OMVs and E. coli after IP, using the PAD4 antibody. [file Image_1.TIF]

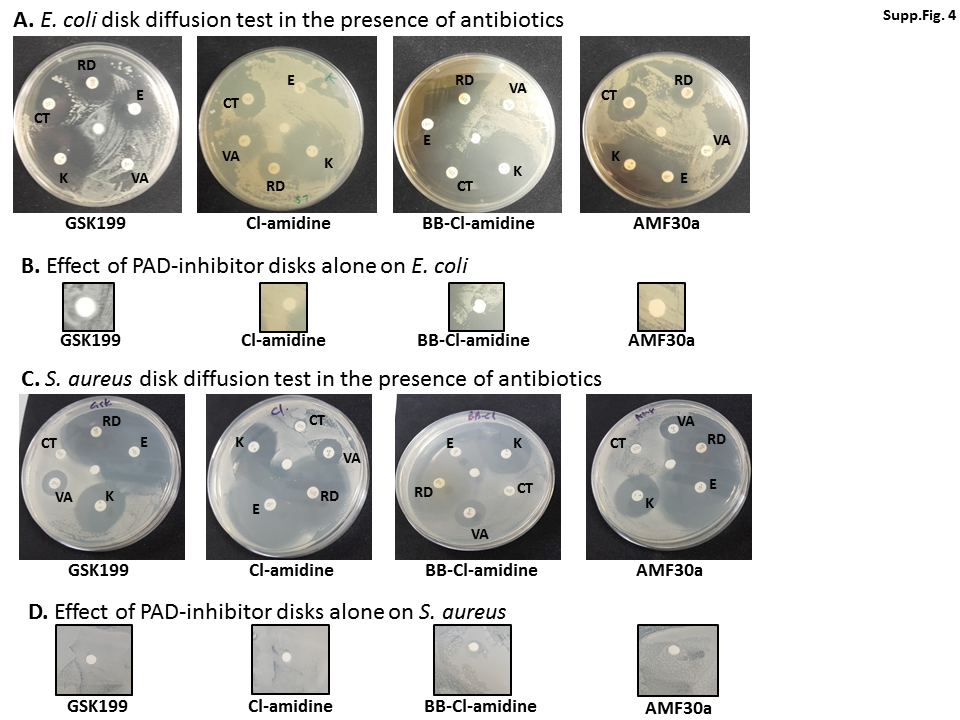

Supplement: Supplementary Figure 4 — Agar plates showing the Kirby-Bauer disk diffusion test for E. coli VCS257 and S. aureus subsp. aureus Rosenbach. (A) Disk diffusion test for E. coli VCS257. (B) Effects of PAD inhibitor disks alone on E. coli are shown. (C) Disk diffusion test for S. aureus subsp. aureus Rosenbach. (D) Effects of PAD inhibitors alone on S. aureus are shown. Disks containing the following antibiotics are indicated: erythromycin (E), vancomycin (V), Rifampicin (R), kanamycin (K), colistin (C). PAD inhibitors used were GSK199 (10 μM; PAD4 inhibitor), Cl-amidine (50 μM; pan-PAD inhibitor), BB-Cl-amidine (5 μM; pan-PAD inhibitor), AMF30a (5 μM; PAD2 inhibitor). [file Image_2.TIF]
